# Recent Developments of Engineered Translational Machineries for the Incorporation of Non-Canonical Amino Acids into Polypeptides

**DOI:** 10.3390/ijms16036513

**Published:** 2015-03-20

**Authors:** Naohiro Terasaka, Yoshihiko Iwane, Anna-Skrollan Geiermann, Yuki Goto, Hiroaki Suga

**Affiliations:** 1 Department of Chemistry, Graduate School of Science, University of Tokyo, 7-3-1 Hongo, Bunkyo-ku, Tokyo 113-0033, Japan; E-Mails: n_terasaka@chem.s.u-tokyo.ac.jp (N.T.); iwane@chem.s.u-tokyo.ac.jp (Y.I.); geiermann@chem.s.u-tokyo.ac.jp (A.-S.G.); y-goto@chem.s.u-tokyo.ac.jp (Y.G.); 2 Japan Science and Technology Agency, Core Research for Evolutional Science and Technology, University of Tokyo, 7-3-1 Hongo, Bunkyo-ku, Tokyo 113-0033, Japan

**Keywords:** tRNA, ribosome, non-canonical amino acids (ncAAs), aminoacyl-tRNA synthetase (AARS), ribozyme, genetic code expansion, genetic code reprogramming

## Abstract

Genetic code expansion and reprogramming methodologies allow us to incorporate non-canonical amino acids (ncAAs) bearing various functional groups, such as fluorescent groups, bioorthogonal functional groups, and post-translational modifications, into a desired position or multiple positions in polypeptides both *in vitro* and *in vivo*. In order to efficiently incorporate a wide range of ncAAs, several methodologies have been developed, such as orthogonal aminoacyl-tRNA-synthetase (AARS)–tRNA pairs, aminoacylation ribozymes, frame-shift suppression of quadruplet codons, and engineered ribosomes. More recently, it has been reported that an engineered translation system specifically utilizes an artificially built genetic code and functions orthogonally to naturally occurring counterpart. In this review we summarize recent advances in the field of ribosomal polypeptide synthesis containing ncAAs.

## 1. Introduction

**Figure 1:**
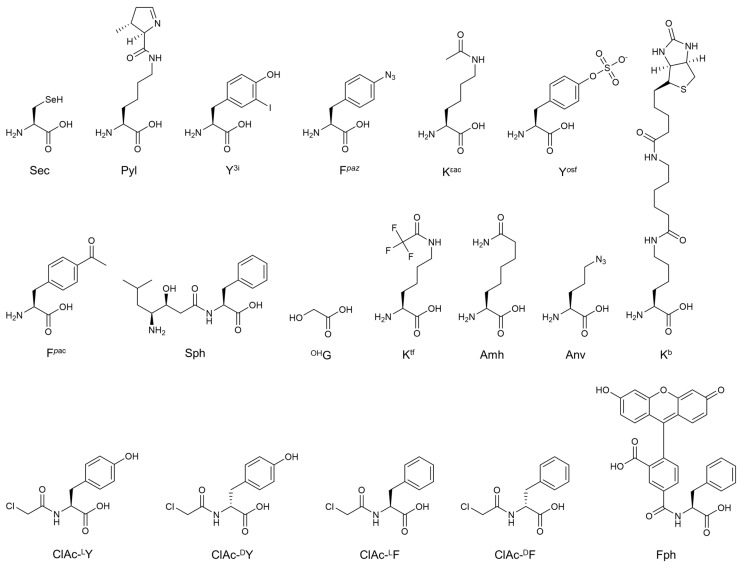
Amino acids described in this review. Sec; selenocysteine, Pyl; pyrrolysine, Y^3i^; 3-iodo-l-tyrosine, F*^p^*^az^; *p*-azido-l-phenylalanine, K^εac^; ε-*N*-acetyl-l-lysine, Y*^o^*^sf^; *o*-sulfo-l-tyrosine, F*^p^*^ac^; *p*-acetyl-l-phenylalanine, Sph; (3*S*,4*S*)-4-amino-3-hydroxy-6-methylheptanoyl-phenylalanine, ^O^^H^G; α-glycolic acid, K^tf^; ε-*N*-trifluoroacetyl-l-lysine, Amh; 2-amino-7-aminocarbonylheptanoic acid, Anv; l-azidonorvaline, ClAc-^l^Y; *N*-chloroacetyl-l-tyrosine, ClAc-^d^Y; *N*-chloroacetyl-d-tyrosine, ClAc-^l^F; *N*-chloroacetyl-l-phenylalanine, ClAc-^d^F; *N*-chloroacetyl-d-phenylalanine, Fph; *N*-(5-FAM)-l-phenylalanine, K^b^; ε-(6-(biotinoyl)amino)hexanoyl-l-lysine.

Assignment of 20 canonical (or proteinogenic) amino acids to trinucleotides, so-called codons, is achieved by specific acylation of tRNA with cognate amino acid catalyzed by aminoacyl-tRNA synthetase (AARS). Since each tRNA has a trinucleotide (anticodon) that pairs with the codon, the codons on mRNA can be decoded by the cognate aminoacyl-tRNAs (AA-tRNAs) according to the genetic code; and thus ribosome is able to catalyze the formation of peptide bond along the mRNA template, to yield a polypeptide with the encoded sequence. Although the genetic code is well conserved in all organisms, some exceptions have been found [1]. The first exception was found in yeast mitochondria that UGA stop codon is reassigned to Trp [2]. More recent study of metatranscriptome analysis has revealed that the extensive UGA and UAG stop codons are reassigned to Trp (or Gly) and Ser (or Gln), respectively, in some bacteriophage, and UGA to Trp (or Gly) in some bacteria [3]. In addition to 20 canonical amino acids, two more amino acids are utilized by ribosomal translation in nature. Selenocysteine (Sec, Figure 1) is co-translationally incorporated into proteins by reprogrammed UGA in all three domains of life [4]. In some archaea and bacteria, pyrrolysine (Pyl, Figure 1) is also incorporated in response to UAG [5,6].

While 22 amino acids are used in the native translation system, it has been demonstrated that hundreds of different non-canonical amino acids (ncAAs) can be incorporated into nascent polypeptide chain by engineering of the genetic code; for instance, those containing not only post-translational modified sidechains found in nature, but artificially designed fluorescent or bioorthogonal functional groups (Figure 1) [7]. Assignment of ncAAs in the genetic code has been achieved by two methodologies: genetic code expansion [8,9] and genetic code reprogramming [10]. The former method generally assigns a ncAA (or multiple ncAAs) to nonsense codon(s), such as stop codons and artificially programmed quadruplet codon(s). Genetic code expansion has been applied for both *in vivo* and *in vitro* expression of proteins with ncAAs. In the latter method multiple sense codons are simultaneously reassigned with ncAAs. Genetic code reprogramming has been dominantly utilized in *in vitro* reconstituted translation system to produce short polypeptides containing diverse exotic ncAAs such as d-amino acids and *N*-methyl-amino acids.

**Figure 2:**
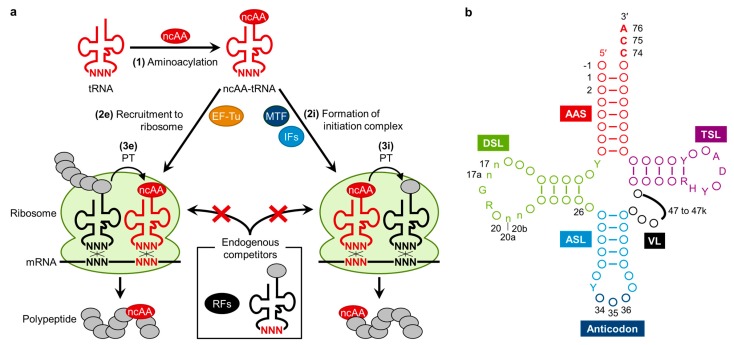
(**a**) General scheme for ribosomal incorporation of non-canonical amino acids (ncAAs) into polypeptides. (1) Engineered tRNA charged with ncAA is prepared by chemical synthesis, aminoacyl-tRNA synthetase (AARS), or ribozymes. (2e) The ncAA-tRNA is recruited to the ribosome by EF-Tu during elongation event. (3e) Peptidyltransfer (PT) reaction between peptidyl-tRNA at the P site and ncAA-tRNA at the A site is catalyzed by ribosome. In engineering of initiation, (2i) ncAA-tRNA^fMet^ is involved in an initiation complex by initiation factors (IFs) and (3i) the first peptide bond is formed by PT reaction; (**b**) Clover leaf structure of tRNA [11]. Conserved bases are described, circles represent non-conserved bases, and numbers indicate the nucleotide position. Positions 34 to 36 (dark blue) corresponds to the anticodon and position 74 to 76 (red) are universally conserved as CCA. The sequences composing the intron and the extra bases presenting in the variable loop (position noted 47 to 47k) are shown as black line. AAS (magenta), the amino acid-accepting stem; DSL (green), the dihydrouridine stem and loop; ASL (cyan), the anticodon stem and loop; VL (black), the variable loop; TSL (purple), the thymidine stem and loop; Y, pyrimidine; R, purine; H, not G; D, not C. The n bases at position 17, 17a, 20a and 20b are optional bases not present in all tRNAs. Position-1 bases are found in all cytoplasmic mature tRNA^His^_GUG_ from the three biological domains.

In general, three events are critical for incorporation of ncAAs into polypeptide (Figure 2a). (1) Aminoacylation: a ncAA needs to be charged onto a tRNA bearing an anticodon corresponding to the objective codon, resulting in the formation of ncAA-tRNA. (2e) Recruitment to ribosome: the ncAA-tRNA is required to bind to EF-Tu (elongation factor Tu) and go to the ribosome A site, and then forms base pairs between tRNA anticodon and mRNA codon. (3e) Peptidyltransfer (PT): peptide bond formation between the peptidyl-tRNA at the ribosome P site and ncAA-tRNA at the A site should be catalyzed by ribosome, resulting in the incorporation of the ncAA into the nascent polypeptide chain. ncAAs can be utilized not only in the elongation pathway but also in the initiation pathway when charged onto the initiator tRNA^fMet^. For ncAA incorporation via initiation, (2i) formation of initiation complex with the ncAA-tRNA^fMet^ assisted by MTF (Methionyl-tRNA formyltransferase) and IFs (initiation factors) and (3i) the efficient PT reaction between ncAA-tRNA^fMet^ at the P site and AA-tRNA at the A site are required. Importantly, these engineered events are normally competed with canonical AA-tRNAs and/or release factors (RFs), which can potentially read the codon artificially assigned to ncAA. Thus, complete suppression of such undesired background events is also important for efficient ribosomal synthesis of polypeptides bearing ncAAs. In addition, in the case of ncAAs incorporation *in vivo*, high intracellular concentration of ncAAs is also required.

In this review, we discuss strategies for genetic code engineering, which have enabled efficient incorporation of ncAAs into polypeptide. Moreover, recently reported concepts of genetic code manipulation, which can increase the number of amino acids usable in a translation system are also described.

## 2. Engineering of Translation Components for the Improvement of ncAAs Incorporation Efficiency

The stop codons [12] and quadruplet codons [8,13], which are often used as suppressor codons in genetic code expansion methods, generally suffer from undesired termination and/or incorporation of canonical amino acids, because RFs and endogenous cognate or near-cognate AA-tRNAs competitively read these codons [14]. In the case of *in vitro* engineering of translation, simple depletion of competing factors, such as canonical amino acids, AARSs, and RFs from the reaction mixture, has succeeded to suppress the undesired background reactions [10,15,16,17,18]. On the other hand, suppression of the competitions in *in vivo* genetic code expansion has been more demanding.

Although exclusion of RF1 is one way to prevent undesired termination at the UAG stop codon, depletion of the RF1-coding *prfA* gene had been considered to be a formidable challenge in this field since RF1 is an essential factor recognizing the UAG stop codon which is not a terminator for RF2 in prokaryotic translation [19]. Therefore, substitution of the UAG stop codons in genome with other stop codons was expected to be an appropriate strategy to successfully knock out *prfA* gene. Recently, knockout of *prfA* gene has been achieved by several research groups. First, in order to sufficiently express the seven essential genes (*coaD*, *murF*, *hda*, *mreC*, *hemA*, *lpxK*, and *lolA*) naturally bearing UAG stop codons in the absence of RF1, Mukai *et al.* [20] introduced plasmids coding an amber suppressor tRNA and these seven essential genes whose stop codons were substituted with UAA, the terminator for RF2, allowing for establishing a RF1-knockout strain, referred to as RFzero. When an AARS-tRNA pair capable of incorporating 3-iodo-l-tyrosine (Y^3i^, Figure 1) was introduced in the RFzero strain, Y^3i^ was incorporated at as many as six sites of UAG present in GST (glutathione *S*-transferase). Unfortunately, the RFzero strain had a poorer growth rate compared with the wildtype strain. This is probably because the expression of over 300 genes bearing the UAG stop codons was supposedly disturbed by knock-out of RF1 and alternative incorporation of Y^3i^ at the UAG stop codons. In a later report, UAG codon present in a nonessential gene *sucB* that is responsible for the energy regeneration was substituted with UAA codon, and then this engineered gene was supplemented to the above seven essential genes. This treatment significantly improved the growth of RFzero strain [21]. In fact, this new strain has enabled for the incorporation of Y^3i^, F*^p^*^az^ (*p*-azido-l-phenylalanine), or Y*^o^*^sf^ (*o*-sulfo-l-tyrosine) at seven sites in GST as well as K^ε^^ac^ (ε-*N*-acetyl-l-lysine) at four sites in human histone H4 (Figure 1). Furthermore, Lajoie *et al.* [22] substituted all of the 321 UAG stop codons throughout the *E. coli* genome with UAA codons and knocked out *prfA*. Unfortunately, the *prfA*-deficient strain represented slower growth than wildtype possibly due to 355 off-target mutations during the substitutions of UAG codons and the suppression efficiency was similar to wildtype strain when F*^p^*^ac^ (*p*-acetyl-l-phenylalanine, Figure 1) was incorporated at three sites in GFP.

Johnson *et al.* reported another approach based on the hypothesis that the lethality caused by the knockout of *prfA* gene might be due to insufficient termination activity of RF2 to UAA [23]. T246A mutation was introduced in RF2 in order to improve its UAA termination activity [24], and also an in-frame UGA autoregulation element present in the *prfB* gene coding RF2 was eliminated to relieve a potential burden imposed on the expression of RF2. In the resultant strain, RF1 was successfully knocked out; the resulting strain was referred to as JX3.0. In addition to these mutations in JX3.0, a spontaneous A293E mutation in RF2 showed fast-growth phenotype, whose strain was referred to as JX33. In JX33, the incorporation of F*^p^*^ac^ was achieved at ten sites simultaneously in GFP (Green Fluorescent Protein).

For *in vivo* genetic code expansion, sufficient intracellular concentration of ncAAs is also required. Most ncAAs can be accumulated in cells at a high enough concentration for aminoacylation and translation by just adding ncAAs into the medium. However, intracellular level of some ncAAs does not reach enough for incorporation of ncAA into protein without genomic or metabolomics engineering. For example, incorporation of phosphoserine into human MEK1 (mitogen-activated ERK activating kinase 1) in *E. coli* was achieved by deletion of *serB*, encoding phosphoserine phosphatase SerB [25]. To further increase the intracellular concentration of phosphoserine, Δ*serB* cells were grown in low or high phosphate minimal media [26]. In the low phosphate media, the PHO (phosphate) regulon is induced [27], which stimulates the uptake of phosphoserine by PhnE [26]. On the other hand, in the high phosphate medium, the degradation of phosphoserine by PHO regulon is suppressed [26]. Under these optimized conditions, intracellular level of phosphoserine was elevated at a comparable concentration to those of canonical amino acids, and incorporation of phosphoserine into protein was improved. Another example is incorporation of hydroxyproline. In order to increase the intracellular concentration of hydroxyproline in *E. coli*, endogenous low-affinity proline transporters encoded by the *putP*, *prop*, and *proU* genes were upregulated under osmotic stress. This approach increased the uptake of hydroxyproline and enabled ribosomal synthesis of proteins containing hydroxyproline [28].

The availability of ncAA-tRNAs (Figure 2a, event 1) is also a crucial factor to carry out the incorporation of ncAAs into peptide chain. A classical method is the combination of enzymatic tRNA aminoacylation with a chemical modification of the charged amino acid. For example, Phe-tRNA was converted to phenyllactyl-tRNA in the presence of nitrous acid, where the α-amino group was deaminated to the α-hydroxy group [29]. This approach was also applied to prepare *N*-methylation on canonical AA-tRNAs involving three steps; (1) the α-amino group of AA-tRNA was protected using *o*-nitrobenzaldehyde; (2) reductively methylated using formaldehyde; and (3) deprotected by UV radiation to liberate the free α-*N*-methyl-amino group [30]. Another classical approach is the combination of chemical aminoacylation and enzymatic oligonucleotide ligation involving three steps; (1) pdCpA was chemically aminoacylated using an appropriate activated amino acid donor with an *N*-protected group followed by HPLC purification; (2) ligated to tRNA lacking the 3'-terimnal CA by means of T4 RNA ligase; and (3) deprotected to liberate the free α-amino group [31,32]. Although these methods have been utilized and are in principle applicable to nearly unlimited kinds of ncAAs, they are technically demanding and laborious; thereby difficult to perform consistently well for various ncAAs.

When the genetic code expansion is performed *in vivo*, availability of ncAA-tRNA generally relies on an exogenously introduced AARS specifically paired with an orthogonal tRNA. The orthogonal tRNA should be inert to endogenous AARSs. At the same time, the exogenous AARS should be engineered to charge the ncAA onto the orthogonal tRNA, but not onto endogenous tRNAs. Several orthogonal AARS–tRNA pairs have been successfully developed [7], and thus far demonstrated expression of proteins containing one or two kinds of ncAAs. However, the choices of usable ncAAs are yet limited to a few subgroups, because the majority examples of the engineered AARSs are based on *Methanococcus jannaschii* TyrRS [9], *Methanosarcina barkeri* PylRS [33,34], or *Methanosarcina mazei* PylRS [35,36]; therefore, the usable ncAAs for these mutant enzymes are limited to Phe or Lys analogs.

An alternative method using tRNA aminoacylation ribozymes, known as flexizymes, has been devised and applied to both expansion [37] and reprogramming of the genetic code [18] *in vitro*. There are three types of flexizymes (dFx, eFx, and aFx) [38,39,40] and these flexizymes can charge a wider range of ncAAs esterified with cognate leaving groups, such as *N*-methyl-amino acids [41,42,43], *N*-alkyl-glycines [44], cyclic *N*-alkyl amino acids [45], *N*-acyl-amino acids [46], exotic peptides [47], α-hydroxy acids [42,48], and d-amino acids [49], onto tRNAs. By means of these flexizymes, we are able to prepare a wide range of ncAA-tRNAs regardless of the body and anticodon sequences since they recognize only the CCA-3' end of tRNAs by base pairs (Figure 2b) [50,51,52]. Importantly, the integration of the flexizyme technology with a custom-made reconstituted translation system, referred to as FIT (Flexible *In-vitro* Translation) system [18], enables us to readily reprogram the genetic code, and thus allows us to express nonstandard peptides containing not only multiple ncAAs but also natural product-like macrocyclic and *N*-methylated backbone scaffolds (see the sections below for more discussions). Although the application of the flexizyme technology is thus far limited to *in vitro* experiments, it has given various opportunities to reprogram the genetic code with great ease (*vide infra*).

The EF-Tu-mediated delivery of ncAA-tRNA also plays a critical role in determining the efficiency of ncAA incorporation into nascent peptide chain (Figure 2a, event Figure 2e). EF-Tu likely binds some ncAA-tRNAs weaker than canonical AA-tRNAs, especially those bearing sterically bulky sidechains [53,54] or highly negatively charged sidechains [55]. To enhance the affinity of EF-Tu to ncAA-tRNAs, both EF-Tu and tRNA have been engineered. The amino acid binding pocket of EF-Tu has been engineered to accommodate non-canonical side chains of some ncAAs whose side chains are large aromatic groups [54] and phosphoserine [25,56]. Uhlenbeck *et al.* have reported that certain species of tRNAs have higher affinities to EF-Tu than other species of tRNAs in order to compensate the difference in affinity of amino acid sidechains, *i.e.*, making a uniformed balance of affinities for all canonical AA-tRNAs to EF-Tu for efficient delivery [57]. In fact, it was recently reported that engineering of the tRNA body sequence enabled to increase the affinity of ncAA-tRNA to EF-Tu, *e.g.*, incorporation of a ε-(6-(biotinoyl)amino)hexanoyl-l-lysine (K^b^, Figure 1) was improved using tRNA^Ala^ compared to that when using tRNA^Phe^ [58]. Likewise, engineered tRNA^Glu^ (termed as tRNA^GluE2^) has improved the efficiency of incorporation of some ncAAs compared with the orthogonal tRNA^Asn^ (termed as tRNA^AsnE2^) previously used for many genetic code reprogramming experiments [51].

**Figure 3:**
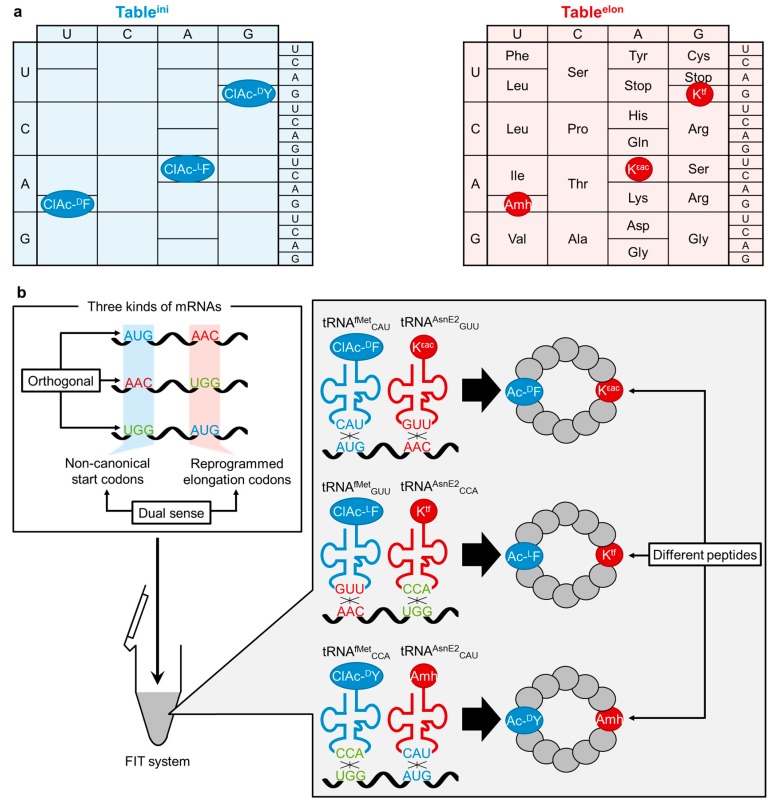
Coexpression of cyclic peptides bearing various ncAA initiators and ncAA elongators. (**a**) The initiation table (table^ini^, blue) designates multiple initiators while the elongation table (table^elon^, red) designates various ncAAs as well as cognate amino acids. Some of codon triplets are dual sense, *i.e.*, designating different amino acids in table^ini^ and table^elon^; (**b**) Schematic illustration of the coexpression. Blue and red tRNAs indicate initiator tRNAs^fMet^ and elongator tRNAs^AsnE2^, respectively. The chloroacetylated ncAA initiators react post-translationally with a downstream Cys residue to form macrocyclic structures.

Engineering of ribosome has been also attempted to improve the recruitment of ncAA-tRNA to ribosome and peptidyltransfer reaction involving ncAAs (Figure 2a, event Figure 2e and Figure 3e). For instance, engineering of 16S rRNA has successfully improved the incorporation efficiency of Sec [59], decoding efficiency of UAG stop codon [60], or quadruplet codons [61]. It has been also reported that incorporation efficiency of d-amino acid and β-amino acids at the UAG codon could be enhanced by certain mutations of the peptidyl transferase center (PTC) in 23S rRNA [62,63,64]. On the other hand, it has been recently reported that the wildtype ribosome was able to incorporate a nearly dozen of d-amino acids in the FIT system [65]; therefore, suppression of the competing background (*vide supra*) and the aforementioned tRNA engineering could improve d-amino acids more significantly than previously thought. Since the aforementioned mutant ribosomes were able to enhance the incorporation of d-amino acids even under the conditions where competing events, including misincorporation mediated by endogenous AA-tRNAs and termination mediated by RFs originating from the S30 extracts were present, it is of interest to see if the combination of mutant ribosomes in the FIT system, where the undesired competing backgrounds are suppressed, will further enhance their incorporation efficiency in future experiments.

## 3. Engineering of tRNA^fMet^ for Usage of Multiple Initiators and Initiation Codons

In the genetic code, each codon generally assigns a single kind of amino acid or termination. However, AUG exceptionally assigns two amino acids, α-*N*-formylmethionine (fMet) and Met, for initiation and elongation, respectively in bacteria [66]. This “dual sense” assignment is achieved by two different AA-tRNAs, initiator fMet-tRNA^fMet^_CAU_ and elongator Met-tRNA^Met^_CAU_. fMet-tRNA^fMet^_CAU_ is produced by introducing formyl group to the α-amino group of Met-tRNA^fMet^_CAU_ by MTF (Methionyl-tRNA formyltransferase), where the formylation is critical for recognition by IF2 [67] and rejection by EF-Tu [68]. Formation of the initiation complex of fMet-tRNA^fMet^_CAU_ with IF1–3, mRNA and 30S ribosome subunit allows for recruiting 50S ribosome subunit, which leads to the initiation of translation. The *E. coli* initiator tRNA^fMet^_CAU_ bears some unique structural features distinct from the elongator tRNA^Met^_CAU_; the C1 and A72 in tRNA^fMet^_CAU_ are unpaired as opposed to the G1:C72 pair in tRNA^Met^_CAU_, which is the most important determinant for MTF; A11:U24 in the stem of DSL (the dihydrouridine stem and loop) as opposed to C11:G24, which is possibly important for favorable structure of DSL for MTF; and three consecutive G:C base pairs in the stem of ASL (the anticodon stem and loop), which are crucial to IF3-dependent tRNA^fMet^_CAU_ discrimination (Figure 2b) [69,70]. These distinct features determine the specific interaction with IFs and fMet-tRNA^fMet^_CAU_ over Met-tRNA^Met^_CAU_ [71].

While fMet is the only initiator in the native translation system, engineering of initiation has achieved incorporation of non-fMet amino acids into the N-terminus of nascent peptide chain. A classical approach for this engineering relies on the substitution of the anticodon CAU of tRNA^fMet^ to other triplets. Because the tRNA aminoacylation activity of some AARSs, such as ValRS, is dictated by the recognition of anticodon over body sequences, these AARSs are able to mis-aminoacylate tRNA bearing their cognate anticodons albeit the body sequence remains the same as tRNA^fMet^. Moreover, MTF catalyzes α-*N*-formylation on the amino acid charged onto tRNA^fMet^ regardless of anticodon sequences. Thus, tRNA^fMet^_GAC_, for instance, could be mischarged with Val followed by α-*N*-formylation to yield fVal-tRNA^fMet^_GAC_ [72,73]. This fVal-tRNA^fMet^_GAC_ could act as an initiator of translation for an mRNA template containing GUC “initiator” codon instead of AUG. However, this method is only applicable to some AARSs, such as GlnRS and PheRS, capable of tolerating the alteration of tRNA body sequence from the cognate to tRNA^fMet^, and the repertoire of usable initiators is certainly limited to the canonical AAs.

Reprogramming of the initiation event by means of the FIT system has overcome this limitation [46]. The flexizyme technology enabled for the preparation of not only the canonical 20 AA-tRNA^fMet^_CAU_ molecules but also a wide variety of ncAA-tRNA^fMet^_CAU_. When the FIT system lacking Met was utilized in the presence of the AA- or ncAA-tRNA^fMet^_CAU_, the ribosomal peptide synthesis was initiated with the designated AA as well as ncAA. Notably, when the free α-amino group in the canonical AA-tRNA^fMet^_CAU_, the *N*-terminus of the expressed peptides is formylated. Although initiation with d-amino acids charged onto tRNA^fMet^_CAU_ was significantly poorer than l-amino acids, this was attributed to poor formylation on the α-amino group of d-aminoacyl-tRNA^fMet^_CAU_ catalyzed by MTF [49]. It turned out that preacylation, such as acetylation, significantly enhanced the efficiency of initiation with not only canonical AA but also ncAA including d-amino acid [49]. Such empirical knowledge has been applied to express peptides with various *N*-terminal group, d-amino acids [49], fluorescent/biotin-labeled amino acids [51,74,75,76], and exotic peptides [47,77] (Figure 2a, Figure 2i and Figure 3i). It should be noted that the combination of peptide expression initiated with γ-amino-dipeptides, e.g., statine-phenylalanine (Sph, (3*S*,4*S*)-4-amino-3-hydroxy-6-methylheptanoyl-phenylalanine), and Cys-Pro-^HO^G (^HO^G denotes α-glycolic acid and upon ligation with Pro the ester bond is formed) at the *C*-terminal region enables for the production of the head-to-tail backbone-macrocyclic peptides (*i.e.*, ligation of the *N*-terminus and *C*-terminus via the peptide bond). Although such γ-amino acids are extremely difficult to incorporate into the nascent peptide chain, this methodology produces backbone-macrocyclic peptides containing various γ-amino acids at the middle of sequences [77].

More recently, the “dual sense” assignment seen in fMet and Met on AUG has been expanded to other sense codons [78]. Taking the advantage in flexibility of the substrate choices of the FIT system, various initiator tRNAs^fMet^_XXX_ and elongator tRNAs^AsnE2^_XXX_ were prepared, where XXX represents anticodon triplets of choice that assign *N*-acyl-ncAA and ncAA whose sidechains could be different. Note that the newly assigned dual sense codons could be simultaneous used (Figure 3a), thus allowing us to assign multiple initiators in the initiation table (table^ini^) and also multiple elongators in the elongation table (table^elon^). The report [78] demonstrated one-pot coexpression of macrocyclic peptides based on three dual sense codons, AUG, AAC, and UGG, where three *N*-chloroacetylated ncAA initiators with different configurations and side chains (ClAc-^d^F, ClAc-^l^F and ClAc-^d^Y, see Figure 1 for their structures) were assigned to the table^ini^ while three ncAA elongators (K^εac^, Amh and K^tf^) and 17 canonical amino acids were designated in table^elon^ (Figure 3a). Three mRNAs, each of which contains a noncanonical start codon and a reprogrammed elongation codon distinct from those on other mRNAs were simultaneously translated in the FIT system (Figure 3b). These dual sense codons were orthogonally decoded by their cognate ncAA-tRNAs^fMet^ and ncAA-tRNAs^AsnE2^, yielding three peptides bearing different *N*-chloroacetylated ncAAs at the *N*-terminus. The *N*-terminal chloroacetyl group post-translationally undergoes spontaneous cyclization with a downstream Cys residue [46]. Consequently, three macrocyclic peptides bearing different ring-closing structures and ncAAs were produced in a translation system. This study clearly showed that the “adaptor hypothesis” [79,80], which highlights the importance of codon–anticodon interactions in accurate mRNA decoding, can be extended to both initiation and elongation events under reprogrammed genetic code involving the dual sense codons. In addition, this methodology can expand the repertoire of initiators and structural diversity of peptides simultaneously synthesized in one translation mixture. Thus, such an expression system can be applied for the discovery of bioactive non-standard peptides using mRNA-encoded non-standard peptide libraries [81]. Indeed, a random cyclic peptide library containing ^d^Y and K^tf^ designated by a dual sense AUG codon was constructed by a FIT system, and cyclic peptides armed with a mechanism-based warhead (K^tf^) that selectively inhibit NAD (nicotinamide adenine dinuclotide)-dependent deacetylase sirtuin-2 were successfully developed [82].

## 4. Engineering of CCA-3' End of tRNA and rRNA for an Orthogonal Translation Machinery

All approaches to genetic code reprogramming by means of the FIT system relies on the use of wildtype ribosome. Because wildtype ribosome uses both AA-tRNAs and ncAA-tRNAs, the engineered genetic code is unusable as the orthogonal code to naturally occurring genetic code; *i.e.*, the present FIT system does not fully function in parallel to the wildtype system. To create such an orthogonal FIT system, Terasaka *et al.* have reported a new pair of engineered ribosome and tRNAs [51].

**Figure 4:**
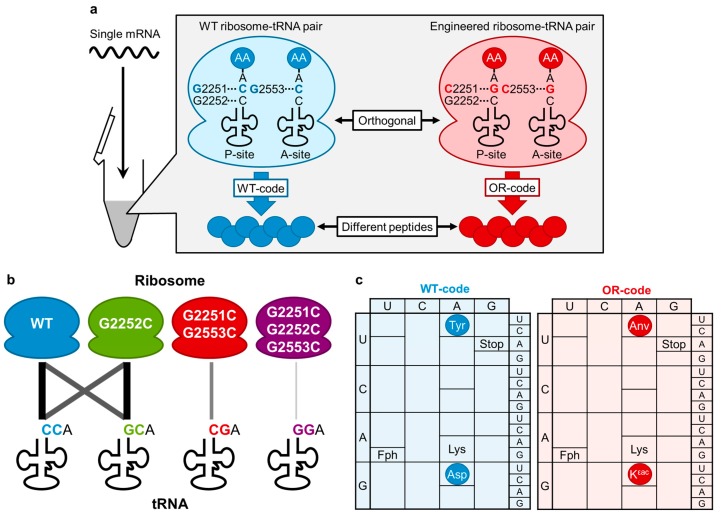
An orthogonal ribosome–tRNA pair via engineering of the PTC. (**a**) Schematic illustration of simultaneous expression of two different peptides by orthogonal ribosome–tRNA pairs. The engineered ribosome–tRNA pair (red) has compensatory mutations (C75G in tRNA, G2251C and G2553C in 23S rRNA) in the PTC. Wildtype ribosome–tRNA pair (blue) and engineered pair are mixed with one single mRNA in an *in vitro* reconstituted translation mixture. Each pair generates two different peptides (blue and red) according to the wildtype (WT)- and orthogonal (OR)-codes, respectively; (**b**) Illustration of compatibility and orthogonality of the ribosome–tRNA mutant pairs. Line thickness indicates the compatibility of translational activity between each ribosome–tRNA pair; (**c**) Two genetic codes designed for simultaneous expression of two different peptides from a single mRNA. Wildtype code (WT-code, blue) comprises the wildtype ribosome–tRNA pair, and OR-code (red) comprises the G2251C/G2553C-ribosome–tRNAs-CGA pair.

In the peptidyl-transferase center (PTC) of *E. coli* ribosome, Watson–Crick base pairs occur between the universally conserved 3' end of tRNAs (C74 and C75) and *E. coli* 23S ribosomal RNA (rRNA) G2251 and G2252 at the P site as well as G2553 at the A site (Figure 4a) [83,84,85,86,87,88,89,90]. Using an analogue of AA-tRNA fragment (C75 mutant puromycin derivatives of the form NPm) as an A-site substrate, Kim and Green [91] reported that the wildtype ribosome preferred the wildtype substrate (CPm) by about two- to five-fold relative to the other substrates (APm, GPm and UPm). Furthermore, G2553C ribosome preferred the compensatory mutated substrate (GPm) by at least 20-fold relative to the other three substrates. These results indicated that the G2253C ribosome-GPm pair could be orthogonal to the wildtype ribosome-CPm pair in peptidyl transfer reaction.

Although the study described above suggests the possibility for the engineering of PTC to develop orthogonal ribosome–tRNA pairs, it was yet unknown how much impact the mutation (or mutations) of 23S rRNA and CCA-3' end of AA-tRNAs in the PTC would give the whole translation reaction to produce polypeptides, including other steps such as accommodation, decoding and translocation. Thus, it would be critical to prepare all possible combinations of the pairs and mispairs of the mutants of ribosome and tRNAs and reinvestigate their whole translation activity [51].

Since most AARSs interact with the universally conserved CCA-3' end [92,93], this hampers charging of amino acids onto tRNAs bearing mutations at the CCA-3' end [94,95,96]. Instead, the flexizymes are the most suitable catalysts for the preparation of AA-tRNAs (as well as ncAA-tRNAs) bearing a mutation or mutations at the CCA-3' end [38,52] because they recognize only the tRNA CCA-3' end by base pairing [50,52]. In fact, the engineered flexizymes containing appropriate compensatory mutations of the tRNA binding sequence were able to efficiently aminoacylate the mutant tRNAs, as opposed to the mispaired flexizymes, which were not [51].

In order to evaluate the activity of mutant ribosomes toward the individual AA-tRNA mutants, the custom-made FIT system [18] was used to produce peptides in the presence of Fph-tRNA^fMet^ and four elongator AA-tRNAs bearing mutations at the CCA-3' end (Figure 1). The studies have revealed that (1) wildtype and G2252C ribosomes crossreact with the tRNA-CCA and tRNA-GCA (*i.e.*, they are not orthogonal systems), while tRNAs-CGA and tRNAs-GGA were inactive (Figure 4b); (2) G2251C/G2553C and G2251C/G2252C/G2553C ribosomes only react with cognate AA-tRNA mutants and do not crossreact with noncognate AA-tRNA mutants, *i.e.*, they are orthogonal systems to the wildtype ribosome (Figure 4b); and (3) although the G2251C/G2252C/G2553C ribosome is active, its activity is significantly lower than the G2251C/G2553C ribosome.

Because the G2251C/G2553C ribosome and tRNAs-CGA pair exhibited only a modest reduction of translation efficiency yet acted as an orthogonal translation system to the wildtype system, we expected that two distinct peptides could be expressed from a single mRNA template under artificially programmed genetic codes (Figure 4a). Fph, Lys, Tyr and Asp were assigned to AUG, AAG, UAC and GAC codons, respectively, in the wildtype code (WT-code, Figure 4c), whereas Fph, Lys, Anv and K^εac^ (Figure 1) were assigned in the orthogonal code (OR-code, Figure 4c). Combining both ribosome–tRNA pairs yielded the desired two peptides from a single mRNA template according to the WT- and OR-codes (Figure 4a). The most important point is that no hybrid products generated from potential crossreading(s) of codons in the non-cognate genetic code were detected, which indicates that these two coexisting translation machineries acted orthogonally and used only their cognate genetic codes.

This study has established a novel approach for genetic code reprogramming, and also demonstrated the importance of interactions between the rRNA and the tRNAs in translation. These results open a new door to opportunities of *in vitro* synthetic biology involving the engineering of the genetic codes and translation machineries.

## 5. Conclusions and Perspectives

As discussed above, the methodologies of ncAA incorporations into polypeptides have given various applications in chemical biology. Non-standard macrocyclic peptides are useful scaffolds for developing drug leads and co-crystallization molecules against protein targets [81,97,98]. The mRNA-based selection system integrated with the methodology of genetic code reprogramming, such as the RaPID system [99], is an ideal platform technology to achieve such goals. Probing the interaction sites of receptor proteins and cognate ligands could also be largely facilitated by the incorporation of photo reactive ncAAs into these proteins [100,101,102]. We expect that more sophisticated applications will be increasingly seen in the future.

On the other hands, we still need to improve the systems currently available for *in vivo* and *in vitro* incorporation of ncAA into polypeptides. *In vivo* genetic code expansion systems, we want to have more diverse kinds of ncAAs for the incorporations and more than two kinds of ncAAs. Most importantly, it is critical to demonstrate unique applications, which cannot be realized by any other methods, for biochemical and biomedical potentials. For *in vitro* genetic code reprogramming, the most advanced FIT system allows us to incorporate a wide variety of ncAAs into peptides, but consecutive incorporations of d-amino acids are thus far unachieved. This likely requires more engineering of EF-Tu, ribosome, and/or tRNAs. More challenges are available for generating efficient orthogonal translation systems that function in parallel to the wildtype system. Likely, these are not so easy to achieve; but if achieved there would be more opportunities and applications for the field of synthetic biology.
